# Thematic Analysis of Written Exposures in an Integrated Intervention for PTSD and Alcohol Use Disorder Following Recent Sexual Assault

**DOI:** 10.1080/10538712.2026.2673338

**Published:** 2026-06-23

**Authors:** Emily L. Tilstra-Ferrell, Selime R. Salim, Alexandra N. Brockdorf, Stephanie Amaya, Sudie E. Back, Christine K. Hahn

**Affiliations:** a Medical University of South Carolina, Charleston, SC, USA;; b Stanford University, Stanford, CA, USA

**Keywords:** Sexual assault, exposure therapy, alcohol misuse, posttraumatic stress disorder, qualitative analysis, efficient intervention

## Abstract

Sexual assault (SA) is alarmingly prevalent and is associated with increased risk for posttraumatic stress disorder (PTSD) and alcohol use disorder (AUD). Exposure-based treatments are a first-line treatment for PTSD and have potential for implementation early after trauma. However, scarce research has been conducted on the application of exposure following *recent* SA among people with PTSD symptoms and AUD, which is critical to inform the development of effective and tailored early interventions for this population. The present study addressed this gap by using thematic analysis to examine content included in written exposure narratives among 10 adults with recent SA (i.e., in the past 3 months) who received a five-session telehealth-delivered integrated intervention: Skills Training and Exposure for PTSD and Substance Misuse (STEPS). STEPS integrates Written Exposure Therapy for PTSD with cognitive-behavioral skills for AUD to address both concerns simultaneously. Participants demonstrated current symptoms of PTSD and met criteria for AUD. Nineteen themes were identified and organized under five categories: Sensory and Memory Processing, Description of Emotions, Description of Cognitions, Individual-Social Impact, and Reactions to Writing. Findings from this study demonstrate that recent SA survivors with AUD can engage in early exposure-based interventions, such as STEPS, with high protocol adherence (e.g. including sensory details, labeling emotions, expressing cognitions). Results also suggest that interpersonal and sociocultural factors may be important for survivors to process and make meaning of after recent SA.

Sexual assault (SA), defined as sexual touching or penetration that is unwanted or occurs without consent, is a serious public health issue in the United States with an estimated 54.3% of women and 30.7% of men reporting lifetime SA (Basile et al., 2022). SA increases risk for posttraumatic stress disorder (PTSD) and alcohol use disorder (AUD). Indeed, 36% of SA survivors develop lifetime PTSD and 18% develop lifetime AUD compared to only 7.0% and 9.1%, respectively, for no-SA groups (Dworkin, 2020). Further, PTSD commonly co-occurs with AUD (Debell et al., 2014). Research on AUD and PTSD after recent SA is scant, but existing evidence with survivors who seek emergency care after SA suggests that about half report alcohol misuse or a recent heavy drinking episode (Resnick et al., 2012). The early weeks to months after SA may be an ideal time to connect survivors with trauma-focused treatment given their increased contact with formal healthcare systems in this period among individuals who seek formal help (Dworkin & Schumacher, 2018). This window of time soon after SA represents a unique opportunity during which to intervene and prevent long-term distress. Thus, early integrated interventions targeting co-occurring PTSD and AUD have the potential to reduce the downstream negative consequences of SA.

Promising intervention approaches for co-occurring PTSD and AUD exist. Exposure-based therapies, such as Prolonged Exposure ([PE]; Foa et al., 2007), are empirically supported treatments for PTSD that have been applied to populations with AUD (Back et al., 2015). Exposure therapy is based on emotional processing theory (Foa & Kozak, 1986) and may include exposure to the trauma memory (e.g., recounting trauma details), as well as exposure to real-life situations that are safe but may be avoided due to PTSD (e.g., going to the store, sleeping with lights off). Emotional processing of the trauma memory is considered a key predictor of outcomes in exposure-based therapies (Alpert, Hayes, & Foa, 2023). Suggested indicators of emotional processing include initial fear activation during exposure, within- and between-session reduction of fear, and changes in trauma-related cognitions. Changes in trauma-related cognitions, in particular, predict PTSD improvements (Alpert, Hayes, & Foa, 2023; Alpert, Hayes, Barnes, et al., 2023), supporting that engagement with the trauma memory promotes new learning opportunities that change maladaptive beliefs.

Exposure-based treatments have been increasingly integrated with cognitive behavioral therapy (CBT) for substance use. For example, Concurrent Treatment of PTSD and Substance Use Disorders Using Prolonged Exposure (COPE; Back et al., 2015), which integrates PE with CBT for relapse prevention, demonstrated safety and efficacy in reducing PTSD and alcohol use (Persson et al., 2017). Integrated treatments using exposure-based therapy are not only safe but are also more effective in addressing PTSD among those with comorbid AUD than treatment approaches addressing PTSD and AUD separately and integrated treatments that do not focus on trauma processing (Hien et al., 2024). However, integrated treatments are lengthy (e.g., 12 sessions in COPE) and have not been evaluated as an early intervention following *recent* trauma. Thus, understanding how exposure-based integrated interventions for PTSD and AUD can be applied to those who with recent SA remains an important area for research.

Historically, there has been concern about intervening shortly after trauma exposure (McNally et al., 2003); however, research has shown that psychological interventions delivered in the early days and months after SA may prevent the development of PTSD symptoms (Short et al., 2020). Importantly, exposure-based interventions have been safely applied after recent trauma. For example, a modified, three-session version of PE was delivered safely to people who presented to the emergency department within 72 hours of the trauma, and findings showed reductions in symptoms with higher effect sizes among those who experienced rape compared to other trauma types (Rothbaum et al., 2012). Although some providers express concerns that exposure-based therapies may exacerbate symptoms (e.g., Becker et al., 2004), evidence suggests that delivering exposure soon after SA does not worsen symptoms and may indeed mitigate risk for long-term PTSD symptoms. Similarly, women with co-occurring PTSD and AUD can effectively engage and benefit from exposure-based therapies (Persson et al., 2017). However, most exposure-based early intervention studies have excluded individuals with substance use disorders (Rothbaum et al., 2012) and have not addressed co-occurring AUD. Thus, the current study aimed to evaluate the use of an exposure-based therapy delivered as part of an integrated intervention after *recent* SA among survivors with *co-occurring* PTSD symptoms and AUD.

Skills Training and Exposure for PTSD and Substance Misuse (STEPS; Hahn et al., 2025) is a recently developed integrated intervention for co-occurring PTSD and AUD that aims to address this clinical gap. STEPS includes five, one-hour sessions and integrates CBT skills training for substance misuse (Cognitive Behavioral Coping Skills Therapy Manual; National Institute on Alcohol Abuse and Alcoholism [NIAAA], 2003) with Written Exposure Therapy (WET; Sloan & Marx, 2019). WET is a five session, exposure-based treatment for PTSD in which participants write about their most distressing traumatic event for 30 minutes each session and discuss their experiences in writing about the trauma with the therapist. Notably, WET is equally as effective as other lengthier, first-line PTSD treatments; further, WET demonstrates significantly lower dropout rates (6–24%) than other first-line PTSD treatments (36–45%), highlighting its tolerability and acceptability (DeJesus et al., 2024). In STEPS, participants complete 20 minutes of CBT skills training prior to the 30 minutes of written exposures. The skills training focuses on identifying and coping with cravings and identifying behavioral alternatives to alcohol/substance misuse (NIAAA, 2003). Preliminary work indicates that STEPS is safe, feasible, and acceptable among recent SA survivors with co-occurring PTSD and AUD (Hahn et al., 2025). In-depth analyses of exposure transcripts can provide insight into patients’ engagement with treatment (e.g., van Minnen et al., 2002). One recent study evaluated written exposures using an observational coding system for insight into emotional processing (Alpert, Hayes, Barnes, et al., 2023). No study to date, however, has conducted in-depth qualitative analyses of content in written exposures among recent SA survivors receiving integrated treatment for PTSD and AUD.

The current study presents secondary analyses examining the content of written exposure narratives among participants in a pilot trial of STEPS who had experienced recent SA (i.e., in the past three months) and had co-occurring PTSD symptoms and AUD. Thematic analysis (Braun & Clarke, 2006) was used to conduct an in-depth qualitative examination of the written exposures to provide qualitative information about trauma processing during treatment. Our aim was to characterize aspects of trauma processing in written exposures after recent SA in line with emotional processing theory (e.g., emotional engagement, changes in trauma-related cognitions) to inform the tailoring of integrated interventions for PTSD and AUD after recent SA.

## Methods

### Participants

We analyzed written exposures from ten treatment-seeking, female adults who experienced a recent SA and participated in a pilot trial of STEPS (Hahn et al., 2023). See Table 1 for demographics. The Institutional Review Board (IRB) at the Medical University of South Carolina approved the clinical trial research from which secondary data were drawn. Participants were engaged in a written informed consent process prior to study activities. Participants were recruited via social media, local community agencies serving recent survivors, and emergency department referrals. Primary inclusion criteria were current alcohol misuse (i.e., score of 3 ≥ on the Alcohol Use Disorder Identification Test – Concise [AUDIT-C], Bush et al., 1998) and acute traumatic stress symptoms related to past three-month SA (i.e., ≥31 on the PTSD Checklist for DSM-5; PCL-5; Blevins et al., 2015). All participants reported past year alcohol misuse prior to the SA (which occurred within 12 weeks of enrollment). Participants were excluded if they indicated current suicidal intent, psychosis, mania, or an eating disorder requiring higher level of care.

### Intervention

All participants received STEPS via telehealth from a licensed clinical psychologist. For details of the STEPS protocol, see Hahn et al. (2023). STEPS is a 5-session integrated intervention for PTSD symptoms and substance misuse following recent SA. Each session, participants receive 20 minutes of CBT skills training for substance misuse prior to completing 30 minutes of written exposures following specific WET session prompts. The CBT skills are adapted from Relapse Prevention: Cognitive Behavioral Therapy Manual for Substance Use Disorders (NIAAA, 2003) and were tailored to SA through a qualitative treatment development process (Hahn et al., 2025). In the current study, participants primarily focused on reducing alcohol use. CBT skills include: psychoeducation about trauma and alcohol/substance use, motivational interviewing and goals setting (session 1); coping with urges and cravings for alcohol/substance use (session 2); managing high-risk thoughts about alcohol/substance use (session 3); assertiveness, alcohol/substance use refusal skills or problem solving selected collaboratively based on patient need/relevance (session 4); and planning for high-risk situations in the future (session 5). The recent SA was used as the index trauma for written exposures for all participants.

Although participants may have had previous experiences of SA, the parent pilot trial aimed to reduce PTSD symptoms after a recent SA and, thus, participants wrote about the most recent assault following prompts outlined in the WET manual (Sloan & Marx, 2019). The writing prompts in sessions one and two focus on writing details of the SA, including what they saw, heard, and smelled during the event, as well as their thoughts and feelings. One participant had difficulty engaging with the initial session prompts during sessions one and two and, therefore, was provided with two extra sessions to ensure they received a full dose of exposure. During session three, participants were encouraged to write about the most upsetting part of the SA and to write about how the SA changed the way they view their life, the meaning of life, and how they relate to other people. Similarly, the writing prompt in session four asked participants to write about the part of the SA that was most upsetting, and to include more details about how the SA impacted their life. Finally, in session five, participants were prompted to write about their thoughts and emotions related to SA and to reflect on how the SA related to their current life and future. Participants completed all five sessions of STEPS.

### Clinical outcomes from parent pilot trial

At baseline, all participants met criteria for past year AUD with severity ranging from mild to severe. Participants indicated consuming an average of 4.43 drinks per drinking day. Four participants (40%) also met criteria for cannabis use disorder, with severity ranging from mild to severe. No other substance use disorders were reported. See Table 1 and the published manuscript (Hahn et al., 2025) for a comprehensive review of mental health outcomes from the parent study from which the present qualitative analyses were derived. To summarize, at the one-month follow-up visit, PCL-5 scores were significantly lower than baseline (*p* < .001; see Table 1) and no participants met the cutoff for probable PTSD (i.e., PCL-5 scores > 31). Compared to baseline, participants also reported significantly fewer drinks consumed on drinking days at the one-month follow-up visit (*p* < .01; see Table 1).

### Procedures for secondary data analysis

For the current study, the research team consulted with a representative at the Medical University of South Carolina IRB who confirmed that analyzing and presenting deidentified quotes from the written exposures obtained during the larger clinical trial was included in the informed consent form completed by participants as part of the larger study. In an abundance of caution and respect for participant confidentiality and the sensitivity of the content in the writing, we also called the 10 participants included in these analyses to obtain additional verbal permission to utilize longer quotes. We only present longer quotes from written exposures of survivors who were contacted, informed of this prospective publication, and agreed for their specific longer quotes to be used. For the remaining participants, we only present brief (less than one sentence), de-identified phrases from the written exposures. Participants wrote with pen and paper and uploaded images of their writing to a secure database, REDCap. An IRB-approved research assistant transcribed the writing for the current analysis. A total of 51 narratives were included (one participant had two extra sessions; another participant only submitted four narratives).

### Data analysis

#### Descriptives and demographics

Demographic data were examined to characterize the sample. SA characteristics and perpetrator details were not uniformly collected in quantitative surveys. Coders tallied SA characteristics described in written accounts related to perpetrator gender, relationship to perpetrator, drug/alcohol facilitation, and revictimization.

#### Thematic analysis

Thematic analysis as described by Braun and Clarke (2006) was conducted following a coding reliability approach (Braun & Clarke, 2021), with the intention to produce a codebook that is consistent and minimizes bias, lending itself for use in future related and replication studies to produce meaningful applied knowledge (Braun & Clarke, 2022). We used an inductive analysis approach, which is data-driven as we did not apply a preexisting framework to the coding process, to identify semantic themes (i.e., identify the explicit meanings of the data). All available written exposure narratives were included in analyses. Two coders (ETF and SS) read all written exposure narratives multiple times to familiarize themselves with the content. Coders then independently generated the initial semantic theme codes. Coders discussed initial codes and discrepancies and developed a codebook comprised of themes and operational definitions. Coders independently coded the same 10% of randomly selected transcripts and interrater reliability (IRR) was calculated using SPSS version 27. IRR following the first round of coding resulted in Cohen’s Kappa value of 0.658. Coders thoroughly discussed discrepancies in coding, revised the themes and subthemes to be more cohesive and parsimonious, and clarified operational definitions in the codebook. Two subsequent rounds of coding 10% of the narratives were completed until yielding a high IRR (Round 2 IRR = .778; Round 3 IRR Kappa = .851). Kappa agreements of .80 or above are considered to indicate strong agreement (McHugh, 2012). Coders discussed the minimal remaining coding discrepancies and finalized the codebook. Coders split all data in half and coded all written exposure narratives following the final codebook. A report of the final themes and subthemes was constructed in a narrative fashion; illustrative quotes were selected to exemplify themes. Longer quotes were condensed, combined and/or blended across participants to ensure participant confidentiality.

## Results

### Sexual assault characteristics

All participants described the perpetrator(s) as male. Among the nine participants who wrote about their relationship with the perpetrator, five were strangers, three were acquaintances, and one was an intimate partner. One person did not write about the relationship to the perpetrator, but identified them as a stranger on their baseline measures. Seven participants wrote about alcohol or drug use during or prior to the SA. Four participants additionally wrote about previous SAs (one child SA, three adult SAs).

### Thematic analysis results

Nineteen themes were identified through thematic analysis. Given the large number and rich nature of themes, we then organized the themes into five higher-level categories: Sensory and Memory Processing, Description of Emotions, Description of Cognitions, Individual-Social Impact of the SA, and Reactions to Writing. We describe the themes as they fall within each broader category; theme names are presented in bolded italics and capitalized below. We report theme prevalence by participant (i.e., number of participants who endorsed each theme across all sessions; see Table 2 for themes by session and Table 3 for operational definitions).

### Sensory and memory processing

All participants wrote about sensory and memory experiences, participants described *Senses (n = 9, 90%)* including the lighting, things that they saw nearby, sounds (e.g., song on the radio), smells (e.g., perpetrator’s cologne), and sensations (e.g., temperature). All five senses (touch, sight, sound, hearing, and smell) emerged across written exposures. Descriptors of these senses are suggestive of high engagement with the prompts and activation of the trauma memory. Over half of participants described *Memory Gaps (n = 6, 60%)*, or that their ability to process specific details of the SA was constrained by difficulty remembering aspects of the event related to the effects of alcohol/drugs or being asleep. In these cases, participants wrote about aspects of the SA that they did remember (e.g., waking up and realizing they were assaulted). Participants also wrote about *Bodily Harm (n = 7, 70%)*, including sustaining physical marks or injuries during the assault, such as bruising, vaginal pain, bleeding, and burning sensations. One person explicitly described fearing for their life during the SA.

### Description of emotions

Consistent with the goal of processing their deepest feelings, all participants expressed a *Range of Emotions (n = 10, 100%)* during and following the SA, which is expected in the context of prompts which ask participants to describe their emotional reactions. Emotions were frequently included in writing for all five sessions; each participant described emotions currently occurring during the written exposures. Participants used words to describe how they felt during the assault which ranged from fear-based emotions (e.g., scared, anxious, afraid, nervous, horrified, scared, helpless), shame (e.g., dirty, used, mortified, disgusted), degradation (e.g., violated, used, vulnerable), and confusion (e.g., bewildered, shocked), to feelings of heartbreak. Participants also described emotions following the SA that were similar to during the SA (e.g., fear, confusion, shame, disgust, dirtiness) and described additional emotions ranging from depressive symptoms (e.g., sad, distraught, tired), numbness, shock, vulnerability, and loneliness. Importantly, many participants wrote about feelings of anger or hate in the written exposure narratives (e.g., anger, hate, irritability, resentment, rage), indicating these are common emotions for individuals who have experienced recent SA and may be an important aspect of processing. Feelings of anger/hate were often expressed toward themselves, the perpetrator, and/or the world since the SA (e.g., hating all people who share attributes with the perpetrator).

### Description of cognitions

Several themes were coded when a participant wrote about the thoughts related to the SA. All participants wrote about cognitions at least once during treatment. Participants explored aspects of helpful and less helpful thinking, with six related themes: *Self-Blame*, *Trust*, *Intimacy*, *Concern for Health after SA*, *Counterfactual Thinking*, and *Balanced Thinking*.

Most participants wrote about *Self-Blame (n = 8, 80%)* related to the SA. SA is always the responsibility of the perpetrator; yet many participants blamed themselves for the SA. For example, participants wrote about being able to prevent or stop SA or for drinking alcohol beforehand (e.g., *“Had I drank less would I have been ok?”)*. Others described inaccurate internalized definitions of rape (e.g., holding the misconception that rape only occurs if the survivor physically fights back) and described self-blame due to experiencing a natural “freeze” reaction when the SA occurred (e.g., “*You should have stood up for yourself”)*.

All participants wrote about negative cognitive changes related to *Trust (n = 10, 100%)* including trusting oneself and others after the SA. For example, one participant shared how her trust in all men was broken, resulting in hypervigilance. Other people described how they perceived their trust was broken with all groups of people (e.g., described need for extensive home security system) and explained how this generalized to trust in all situations, even those seemingly unrelated to the SA. For example, a parent wrote about losing trust in all people, such as not allowing people who had no connection to the SA to care for her children. Participants also wrote about how the SA impacted their trust in themselves interpersonally (e.g., *“I don’t trust myself”*).

Almost half of participants wrote about negative ways that the SA impacted their thoughts about *Intimacy (n = 8, 80%)*, including with friends, partners, and themselves. For example, some participants wrote about how the SA impacted their ability to develop intimate relationships with others and difficulty developing deep, meaningful connections (e.g., *“I have a lot of superficial relationships”*). Participants also described how the SA impacted their ability to have consensual sex with partners. It also included feeling disconnected with the person they were before the SA, reporting not knowing themselves, or perceiving themselves to have lost their identity. Two participants (20%) described *Concerns for Health after SA (n = 2, 20%)* specifically related to fears of contracting sexually transmitted infections and pregnancy.

*Counterfactual Thinking (n = 5, 50%)*: that is, thinking about possible alternatives to past events or “what might have been,” presented in half of the written exposure narratives. Participants described wanting to “undo” the event, such as exploring what might have happened if they left at an earlier time or went somewhere else on the day of the SA. Other participants questioned themselves and wondered if they could have prevented the assault. For example, participants wondered if a different outcome would have occurred if they had responded to the perpetrator in a different manner (e.g., fought back, consumed less alcohol). Participants less commonly described counterfactual thoughts in session five written exposures, suggesting these cognitions may have been actively processed in treatment.

Over half of participants paired unhelpful and/or inaccurate thoughts with more *Balanced Thinking (n = 6, 60%)* about the SA. For instance, a participant who reported being blamed by the police for the SA due to living in a certain neighborhood explained how SA could occur in any location and such statements were inaccurate. Participants also used the writing to productively process and think through the facts of the SA, especially during later sessions. For example, survivors wrote about their expression of “no,” described realizing that at the time of the SA they were too intoxicated to provide consent, described the perpetrator’s strength and weight as being significantly greater, and acknowledged how fighting could have increased danger, recognizing that freezing or fawning may have been the best option for survival. Importantly, in writing about these painful thoughts, survivors often ultimately were able to produce accurate thoughts that challenged distortions, including realizing that SA is never the fault of the victim and challenging internalized rape myths that had produced the self-blame.

### Individual-social impact of the SA

Many themes focused on participants discussing the traumatic event in relation to its impact on social interactions, broader sociocultural context, desire for justice, and their personal experiences and identity. These themes reflect ways in which participants integrated their traumatic experiences into their broader social and individual contexts and underscored the ways in which participants coped with and processed the SA to navigate the interpersonal and societal complexities of post-SA experiences.

Most participants wrote about *Disclosure Experiences (n = 8, 80%)* including feeling fearful they would be blamed or disbelieved if they disclosed to formal sources, such as law enforcement, and/or informal sources, such as close friends or family. These fears resulted in real-world consequences. For example, one participant explained that she was reluctant to seek help due to concern she would not be believed (“*Would they believe me or would they hurt me too?*”). Others described not disclosing for fear they would be blamed (e.g., “*I couldn’t tell anyone because it was my fault I was there”)*. Among those who disclosed, participants described a mix of positive, supportive reactions (e.g., “*My friends have been so understanding with me since the assault*.”) and negative reactions (e.g., feeling blamed for the SA when reporting).

Individuals who experienced negative reactions from trusted individuals following initial disclosures wrote about a diminished sense of trust in formal sources. In contrast, some instances in which survivors described supportive reactions to disclosure were followed by descriptions of choosing to seek necessary SA-related healthcare and legal services. The writing provided a platform for participants to organize their thoughts and explore how reactions to disclosure influenced their perception of the SA. For instance, one participant described how receiving support from her male friends after SA facilitated a shift in her previously expressed negative cognitions about trusting men. However, one-third of participants described *Interpersonal Betrayal (n = 3, 30%)* related to the assault. For example, one participant wrote about the traumatic nature of experiencing SA perpetrated by someone they previously thought was a friend. Others shared feelings of betrayal due to seeing bystanders were present during the SA and chose not to intervene. Participants also processed painful feelings of betrayal experienced when family members or others provided unsupportive responses to disclosure.

When describing the impact of SA, many participants shared a *Desire for Justice* (*n = 6, 60%)* including beliefs that the perpetrator would never be brought to justice and perceptions that the justice system would not serve them. For example, one person wrote about how she debated whether to report to the police and balanced a dialectic that she wanted to report and was unsure if she should move forward as she could recall hearing stories from other women who were blamed or not believed when reporting SA. Some participants processed hopelessness surrounding seeking justice, especially when they had past negative experiences. For example, one participant who also had experienced SA in childhood shared that previous experiences when reporting tainted their views of the likelihood that they would receive justice when reporting the adult SA. Others expressed desire for justice while acknowledging that seeking justice may not result in charges and could harm their wellbeing.

Half of the participants wrote about the intersection of their *Identity in Relation to SA (n = 5, 50%)*. For example, participants questioned if the event occurred because of their identity as a woman (“*Rape, sexual assault, sexual harassment, physical abuse, bullying, body shaming … Is this the price of being a woman?*”). Multiple participants were women from minoritized communities (e.g., women of color, sexual minorities). Participants wrote about how this intersection influenced their mental health in complex ways, at times, compounding harm as a function of systemic oppression, while in other cases facilitating posttraumatic growth through their newfound support systems. For example, participants wrote about the mistreatment of women of color and reflected on the intersection of sexism and racism in SA. Another participant wrote about being targeted by the perpetrator due to being gay, “*… since we were gay, he had assumed that we would desire something from him, as if gay implied something was missing*.”

Reflections also highlighted a link between the *Sociocultural Context in Recovery (n = 2, 20%)*, including sociocultural and political events, as it influenced the psychological aftermath of SA, eroded one’s sense of bodily autonomy, and intensified feelings of powerlessness living in a society that is experienced as misogynist and racist. Participants also reflected on broader cultural narratives surrounding SA, such as the disconnect between media depictions of rape and lived experiences, “*What happened to me was nothing like TV … I woke up in my house, not in a ditch … I was not walking in a dark alley*.” Other participants wrote about the impact of ongoing trials that were broadcasted in the news and the difficulty of witnessing negative experiences among highly publicized SA cases in news/media. Sociocultural influences emerged often in later sessions as participants processed beliefs about the world and made meaning of what it meant to be a survivor in a society built upon rape culture.

Participants often wrote about *Behavioral Changes (n = 8, 80%)* that they employed to adapt and cope following SA. Almost everyone shared that they adopted protective strategies to try to restore their sense of safety (e.g., carrying a weapon, not going out, changing appearance or clothes in effort to be less attractive). Some participants discussed how their safety behaviors were protective in the short-term, but ultimately misaligned with their values (e.g., avoiding social interactions helped them feel safe but also hindered meaningful relationships). Participants wrote about the tension between the need to reclaim their immediate safety and the unintended impact of how these changes influenced activities of daily living (e.g., feeling they cannot leave the home and be safe).

Participants also shared about *Using Substances to Cope (n = 4, 40%)*, particularly alcohol, with the SA. For example, participants wrote about increased substance use as a means of coping with SA-related posttraumatic stress and described how alcohol helped them to forget the SA and reduce the intensity of PTSD symptoms (e.g., “*drinking my problems away in the hopes the pain would go with it”)*. Participants also described coping motives for drinking after SA (e.g., “*everything would be alright after I was drunk*”). Others wrote about urges to use substances during the writing process due to the difficulty of writing about the SA.

Many participants described *Posttraumatic Growth (n* = 6, 60%) including channeling their experience into helping other survivors and engaging in advocacy. Participants described that experiencing SA helped them to recognize and identify rape culture and internalized misogyny (e.g., “*I recognize its [referring to rape culture] internalized harm in our society and within myself”)*. Some people also wrote about how surviving SA fostered a sense of empowerment (e.g., “*I want to be a voice for empowerment and support”)*, stronger self-esteem, and a desire for advocacy (e.g., “*I am willing to fight to make a difference*.”).

Several participants described feelings of gratitude and discovered avenues for increased self-worth. Positive shifts in self-perception were often described as relating to actionable steps to improve one’s mental health. For example, one woman shared that she felt more empowered to stand up for herself and other women after the assault. Another individual described how the SA increased her belief in herself and her abilities to protect her children. Most reflections on posttraumatic growth emerged in later sessions, suggesting potential benefits of written exposure in facilitating growth and consistent with the writing prompts for sessions three through five.

### Reactions to writing

Half of participants included *Reactions to Writing (n = 5, 50%)* in the written exposure narratives, including their experiences of avoidance, persistence, and/or difficulty of engaging with the emotionally taxing content. Although some participants initially doubted their ability to do the written exposure (e.g., expressing the difficulty of the exercise), they ultimately reported a sense of purpose in completing the writing exercises (e.g., “*I don’t want to do this. I know I need to*”) as participants reflected on the value of confronting their trauma through the writing.

#### STEPS adaptations considerations.

Together, these themes yield several considerations for future adaptations to STEPS and other integrated PTSD-AUD treatment models when delivered to individuals who have recently experienced SA. See Table 4 for adaptation considerations based on the themes.

## Discussion

Qualitative analyses identified themes in line with emotional processing theory, which in combination with improvements in PTSD symptoms observed following treatment, may suggest that participants were able to engage in trauma processing recently after SA through the written exposures. Most participants described sensory experiences in the initial sessions and a range of emotional experiences throughout their writing, suggesting adherence to the writing prompts. Participants wrote about a variety of trauma-related cognitions, which is an important mechanism of PTSD improvements in exposure-based therapies (Alpert, Hayes, & Foa, 2023). We found fewer counterfactual thoughts and more balanced thoughts and descriptions of posttraumatic growth in the final writing sessions. In addition, about half of participants also described how aspects of their identity and the sociocultural context related to their recovery from SA, even though these were not explicitly requested in the writing prompts. A subset of participants also wrote about using substances to cope with SA-related distress, which may be expected given that all survivors met criteria for AUD. Results suggest that survivors with co-occurring AUD were able to engage in exposure-based writing in the first three months after SA in the current study.

Most survivors (90%) used sensory information in describing the SA in early writing sessions. Survivors were most likely to describe the sensation of touch, which may reflect the nature of SA as a physical act. Sensory details were present despite 60% of participants endorsing some gaps in their memory of the SA. Some memory gaps were related to alcohol and/or drug use prior to the SA, whereas others were related to their being asleep during the SA. Memory gaps can also occur in relation to peritraumatic dissociation (i.e., dissociation during the trauma). Having clear memories of the trauma is a common inclusion criterion for some clinical trials of exposure-based therapies (Schnurr & Lunney, 2015), but our findings suggest that participants were able to complete the writing focusing on what they did recall. Future research should investigate what amount of memory recall is necessary for survivors to benefit from exposure therapies. This would be especially informative for working with SA survivors given that a significant portion of assaults occur in the context of alcohol and other substance use which can interfere with memory consolidation (Chen et al., 2020; Lawyer et al., 2010). Further, future exposure-based integrated intervention protocols should consider greater flexibility in relation to the degree of trauma memory and details about what happened needed for inclusion to participate. One study evaluated whether inclusion of a written narrative in cognitive processing therapy (CPT) among survivors of substance-facilitated SA supported or hindered recovery (Jaffe et al., 2021). Interestingly, although both groups still benefited from treatment, survivors completing CPT without a written narrative had better PTSD treatment gains at 6-months post-treatment than those who completed with a written narrative. Further research is warranted to inform specific recommendations.

Theoretical models of PTSD propose that peritraumatic dissociation is a risk factor for the development of PTSD by inhibiting trauma processing (Bedard-Gilligan & Zoellner, 2012). However, past research has failed to find an association between peritraumatic dissociation and objective measures of memory fragmentation, suggesting instead that peritraumatic dissociation is related to individuals’ subjective perception of their memories (Bedard-Gilligan & Zoellner, 2012). More recent research has demonstrated that patients’ retrospective reports of peritraumatic dissociation decrease over the course of exposure-based therapies (Thompson-Hollands et al., 2021). Thus, peritraumatic dissociation is not a contraindication for exposure-based therapy and clinicians may approach dissociation as a form of avoidance of trauma-related thoughts and emotions (Bedard-Gilligan & Zoellner, 2012).

Survivors described many emotions throughout each session and most narratives were coded for description of emotions. Specific emotions such as shame, disgust, and anger were commonly expressed. Anger is a common experience after trauma (Maercker & Horn, 2013) and may emerge during exposure after recent SA. Because emotional activation is considered necessary for emotional processing of the trauma memory (Foa et al., 2007), high emotional engagement as seen through frequent use of emotion words may have facilitated processing of the trauma in the current sample (Alpert, Hayes, & Foa, 2023). It is unclear whether writing about certain emotions, such as anger and hate, relates to treatment outcomes. Future research is needed to clarify effects of processing specific emotions in the context of recent SA and alcohol misuse, including if and how processing specific trauma-related emotions predicts change in alcohol craving and use.

Various cognitions were also frequently described throughout treatment, including common trauma-related cognitions (e.g., trust, intimacy, betrayal, self-blame). Several of these cognitions are addressed directly in CPT (Resick et al., 2016) and our study indicates that participants wrote about these core trauma-related cognitions even when not directly prompted. In later sessions, fewer participants expressed counterfactual thinking (i.e., wanting to “undo” the event) and more described balanced thoughts and aspects of posttraumatic growth. This pattern suggests that survivors increasingly moved from unhelpful “what if” and “if only” thoughts to more helpful “what is” thoughts. Nevertheless, many participants included negative trauma-related cognitions in their writing during the final session of STEPS, especially related to the impact of SA on trust. Providers may anticipate that the final writing can contain negative thinking, but treatment gains are sustained after termination (DeJesus et al., 2024).

It is important to note that self-blame was expressed by most participants during STEPS. SA is a highly stigmatized experience within society, and this stigma is often internalized in the form of self-blame, which is detrimental to survivors’ wellbeing (Kennedy & Prock, 2018). It is critical for providers working with survivors with comorbid AUD and PTSD to respond to disclosures of SA from an orientation of belief, compassion, and support (End Violence Against Women International [EVAWI], 2021), be nonjudgmental of SA and alcohol use, and explicitly express that survivors are not to blame for the assault (Gagnon et al., 2018). Given the high prevalence of self-blame among SA survivors, balanced thinking skills that challenge these unhelpful beliefs while simultaneously affirming the survivor’s agency over health behavior change may be useful in integrated interventions tailored to this population. For example, providers may highlight the role of the perpetrators’ responsibility to reduce self-blame while also exploring survivors’ alcohol-related expectancies, eliciting survivors’ reasons for change, and reinforcing the long-term benefits of alcohol use reduction.

Although adverse impacts were clearly documented, many survivors also wrote about how they demonstrated resiliency following the SA. Survivors described their desire to heal after SA, including by prioritizing their mental health and wellbeing. Many survivors wrote about positive changes in their relationships after SA, including ways in which their loved ones demonstrated support, belief, and caring after SA. Some participants described a desire for future advocacy for SA prevention and response, including community activism and educating their children on consent and healthy interpersonal boundaries. The presence of these themes is particularly notable as writing prompts do not specifically solicit information on posttraumatic growth; rather, survivors naturally chose to share their experiences of empowerment in response to instructions to write about the impact of SA. Research on posttraumatic growth following SA is sparse, but evidence indicates that survivors can experience aspects of growth in the weeks after a trauma (Fayaz, 2024). Our findings add to this literature by demonstrating aspects of posttraumatic growth among people who experienced recent SA in the past three months, which is notable given the recency of the trauma. Because cognitive processing and disclosure of the trauma are two factors that promote posttraumatic growth (Henson et al., 2021), it is possible that early intervention using exposure after SA may facilitate not just PTSD symptom reduction but also reclamation of positive experiences and recovery. Future integrated intervention research with SA survivors should consider approaching interventions with a strengths-based, resiliency-oriented lens to highlight values and aspects of growth and empowerment throughout treatment, as well as examine whether these aspects of posttraumatic growth are connected to motivation to reduce alcohol use.

### SA prevention and response

Individual mental health treatment following SA is an important aspect of SA response; however, survivors’ written exposure narratives also revealed community avenues for SA prevention and response. Sixty percent of survivors wrote about concerns related to disclosure, including fear that they would not be believed or would be inaccurately blamed for the SA. Unfortunately, these concerns may have been warranted as some survivors wrote about negative responses to disclosure with informal and formal supports. Yet, other survivors described positive and supportive responses from family and friends; such support appeared to facilitate recovery and help some survivors in modifying trauma-related cognitions. These results are consistent with emerging evidence that more positive social interactions might be protective against worsening PTSD symptoms after recent SA (Howe & Dworkin, 2024). Thus, increasing positive social support after SA may be important for the prevention of long-term distress.

Survivors also wrote about their hopelessness and anger related to lack of justice following SA. These perceptions may reflect reasons why many people are hesitant or choose not to report to law enforcement, aligned with national estimates that only 0.2 to 5.2% of SAs result in conviction or incarceration (Lonsway & Archambault, 2012). Moreover, individuals with intersecting identities wrote about how their concern about not being believed or experiencing discrimination in the legal system was compounded by marginalization. These findings underscore difficulties obtaining justice, especially among people with minoritized identities, and the importance of having an outlet in psychotherapy to process these experiences. It is possible that these concerns may have been amplified in our sample of survivors who may be concerned about experiencing negative reactions or consequences related to alcohol use. Lastly, some wrote about rape culture more broadly or about highly publicized media events eliciting distress and painful reminders of their own SA. These findings highlight the need for providers to consider high-risk situations for alcohol craving and use that may be especially common after SA, including negative reactions to disclosure, interactions with law enforcement, and seeing aspects of rape culture reflected in the news. Future research on integrated interventions could explore the application of cognitive-behavioral and relapse prevention skills for managing high-risk situations to help survivors navigate and respond to sociocultural reminders of SA. Further, continued training efforts for law enforcement may be warranted to reduce negative interactions with law enforcement and secondary distress following investigative interviews.

### Limitations and future directions

It is important to note several limitations of this study. Our findings are drawn from a small sample of adult SA survivors from one region of the southeast US who agreed to participate in the parent clinical trials. Survivors were all female and all perpetrators were male. These characteristics are consistent with national estimates that women disproportionately experience SA, whereas men disproportionately perpetrate SA (Basile et al., 2022). Further, over half of perpetrators were strangers in the present sample, which is not reflective of the average SA experience as the majority of SAs are perpetrated by people known to the victim (e.g., friends, partners, acquaintances; Tapp & Coen, 2024). Another important sample characteristic is that all survivors in our study agreed to participate in a mental health treatment study after a forensic exam. Less than half of survivors seek help from formal mental health professionals after SA (e.g., Fleming et al., 2018). Thus, our results may not generalize to survivors who are not represented in our sample (e.g., those who do not seek services after SA, experience SA perpetrated by a known person, and/or male survivors).

It should be noted that, although the writing prompts do not reference sociocultural and identity-related factors, experiences of racism and sexism emerged as notable topics in the written exposure narratives for several participants. Experiences of oppression are recognized as sources of traumatic stress (Holmes et al., 2016), and future research should evaluate whether prompting participants to consider sociocultural factors in exposure therapy for PTSD may be beneficial for outcomes. Further, it is limited to interpret themes related to sensory, emotional, and cognitive details as definitive indicators of emotional processing in written exposure. While these themes are in line with factors identified as important within emotional processing theory (Alpert, Hayes, & Foa, 2023), we cannot fully determine the extent to which the written exposures activated the feared memory in session. Future research may incorporate physiological indicators of arousal and emotional activation during session (e.g., heart rate, skin conductance; Wangelin & Tuerk, 2015) alongside narrative content. It will be important to explore written exposure narratives in larger and more diverse samples of recent SA survivors. SA and perpetrator characteristics should be collected for future studies to examine possible differences in SA characteristics with regard to written exposure content in larger samples. Finally, future research with larger samples should examine whether themes differ across individuals who experience clinically significant reductions in PTSD and/or AUD post-treatment and those who do not.

## Conclusion

This study conducted an in-depth thematic analysis of written exposure narratives among recent survivors of SA who completed an integrated intervention for co-occurring PTSD and AUD. Results suggest that this population was able to engage with written exposure after recent SA as we observed themes in line with emotional processing of the trauma. Results also provide insights into areas for further tailoring integrated interventions for recent SA survivors, including the development of flexible protocols about the degree of memory necessary to engage in STEPS, discussion of possible high-risk contexts for psychological distress and alcohol craving/use after SA, and aspects of resiliency and personal values that could support goals for trauma recovery. Results encourage further research on engagement of recent SA survivors with PTSD and AUD in early integrated interventions with exposure components in larger samples.

## Figures and Tables

**Table 1.** Participant demographics, sexual assault characteristics, and mental health symptoms.

Category	
*Demographic Information*	*M (SD) / %*
Age	30 (8.37)
Race/Ethnicity	*N*
White	7
Black/African American	3
Hispanic or Latino	1
Not Hispanic or Latino	9
Education Level	
Some High School/GED	2
Some College	2
College Diploma (Associate’s Degree or Higher)	6
Employment Status	
Full Time	6
Part Time	1
Unemployed/ No Regular Employment	3
Annual Household Income	
< $35,000	3
$35,000–50,000	2
$50,000–100,000	3
> $100,000	1
Average Time Since SA at Baseline	35
<1 month	5
1–3 months	5
*Mental Health Symptoms*	*M (SD) / %*
PCL-5 Scores	
Baseline	54.4 (12.19)
Final Therapy Session	21.4 (12.15)
One Month Follow Up	14.11 (10.71)
Percent Days Drinking	
Baseline	65%
Final Therapy Session	55%
One Month Follow Up	37%
Average Drinks per Drinking Day	
Baseline	4.43 (2.27)
Final Therapy Session	3.77 (2.65)
One Month Follow Up	3.31 (1.07)

* Note. Baseline and Final Therapy (*n* = 10); One Month Follow Up (*n* = 9).

**Table 2.** Themes by steps therapy session.

Category/Theme	Session 1	Session 2	Session 3	Session 4	Session 5
**Sensory and Memory Processing**					
Bodily Harm	4	4	4	3	1
Senses	7	6	3	3	0
Memory Gaps	6	4	2	3	1
**Description of Emotions**					
Range of Emotions	6	8	7	7	7
**Description of Cognitions**					
Self-blame	4	3	4	2	2
Trust	1	2	6	5	6
Intimacy	0	1	6	4	1
Counterfactual Thinking	0	3	3	4	1
Balanced Thinking	1	2	0	4	5
Concern for Health After SA	0	0	2	0	0
**Individual-Social Impact**					
Using Substances to Cope	1	1	2	0	1
Behavioral Changes for Safety	0	1	2	4	4
Posttraumatic Growth	0	0	2	2	5
Disclosure Experiences	3	4	4	2	5
Interpersonal Betrayal	1	2	1	2	1
Desire for Justice	0	1	0	3	2
Identity in Relation to SA or Recovery from SA	0	1	1	2	1
Sociocultural Context in Recovery	0	0	1	1	1
**Reactions to Writing**	1	0	1	2	1

Note: Codes are not mutually exclusive and could be coded in multiple sessions. Extra session themes were excluded; Sessions one through four (*n* = 10); Session five (*n* = 9).

**Table 3.** Thematic analysis results and theme definitions.

Theme	N (%)	Example Quote
***Sensory and Memory Processing***		
Bodily Harm	7 (70%)	Survivor described physical marks or injuries that occurred due to the SA.
Senses	9 (90%)	Survivor described one or more specific sensations related to the SA (touch, sound, sight, smell, taste).
Memory Gaps	6 (60%)	Survivor described gaps in memory or difficulty remembering aspects of the SA.
***Description of Emotions***		
Range of Emotions	10 (100%)	Survivor described an explicit emotion (e.g., scared, angry, confused) related to the SA.
***Description of Cognitions***		
Self-Blame	8 (80%)	Survivor described thoughts of self-blame about the SA.
Trust	10 (100%)	Survivor described trust in themselves or others as being negatively affected by the SA.
Intimacy	8 (80%)	Survivor described intimacy with themselves or others as being negatively affected by the SA.
Counterfactual Thinking	5 (50%)	Survivor described counterfactual thoughts in an attempt to “undo” the SA, such as questioning their behaviors the day of the SA and/or considering how they could have prevented the SA.
Balanced Thinking	6 (60%)	Survivor described balanced and helpful thoughts that counter unhelpful thoughts and/or positive self-talk that counters thoughts described related to themes of self-blame, trust, and intimacy.
Concern for Health after SA	2 (20%)	Survivor described concern for their physical health after SA (e.g., pregnancy, sexually transmitted infections).
***Individual-Social Impact of the SA***		
Using Substances to Cope	4 (40%)	Survivor described using drugs or alcohol to cope with the SA.
Behavioral Changes for Safety	8 (80%)	Survivor described engaging in conscious behaviors due to the maladaptive belief that their behaviors can prevent future SA (e.g., changing appearance, carrying weapons).
Posttraumatic Growth	6 (60%)	Survivor described positive emotional or behavioral changes following the SA (e.g., helping others, engaging in advocacy work, improved self-esteem, seeking mental health services, changes in parenting strategies, increased self-compassion).
Disclosure Experiences	8 (80%)	Survivor described experiences related to SA disclosure, including supportive and non-supportive responses from informal sources (e.g., partners, family) and formal sources (e.g., police) as well as concerns about choosing to disclose about the SA.
Interpersonal Betrayal	3 (30%)	Survivor described feeling interpersonal betrayal related to the SA (e.g., perpetrator being someone they formerly trusted, bystanders not intervening with the SA).
Desire for Justice	6 (60%)	Survivor described perceptions about a desire to seek justice after the SA (e.g., desire to report).
Identity in Relation to SA or SA Recovery	5 (50%)	Survivor described aspects of identity (their identity or perpetrator’s identity) in relation to the SA or in relation to recovery from the SA.
Sociocultural Context in Recovery	2 (20%)	Survivor described world news and other current events in relation to SA or recovery from SA.
***Reactions to Writing***		
Reactions to Writing	5 (50%)	Survivor wrote about their reactions in the moment to engaging with the writing prompt.

**Table 4.** Results across the five categories in relation to steps adaptations.

Category	Considerations for Adaptation
Sensory and Memory Processing	Participants described vivid sensory and memory experiences despite often reporting memory gaps. Integrated interventions could consider flexible protocols surrounding the degree of memory necessary to participate.
Description of Emotions	Strong negative emotions were common. Integrated interventions could consider discussing specific negative emotions (e.g., shame, disgust, anger) and how these emotions could elicit alcohol craving and use.
Description of Cognitions	Although sexual assault is always the responsibility of the perpetrator(s), most individuals expressed beliefs about self-blame and identified ways in which they tried to change their behaviors to reduce risk for future victimization. Integrated interventions should address these unhelpful beliefs, while also affirming each individual’s agency over positive health behavior change. Evidence of balanced thinking was common. Integrated interventions could apply these skills to support decision-making about alcohol use, as well as highlight the responsibility of the perpetrator(s).
Individual-Social Impact of the SA	Participants shared how sociocultural factors interacted with their trauma recovery. Integrated interventions may want to identify and plan for high-risk situations specific to sexual assault that might trigger PTSD symptoms and craving, such as highly publicized media events involving sexual assault and negative reactions to sexual assault disclosure. Aspects of posttraumatic growth and resiliency arose naturally. Integrated interventions could highlight how these values and strengths might support treatment engagement and alcohol use reduction.
Reactions to Writing	Half of participants shared emotional reactions to exposure. Integrated interventions may benefit from planning strategies to cope with difficult emotions that may arise during treatment and could lead to craving.

## Data Availability

Codebook is available from the corresponding author upon request; data are not made available to protect participant confidentiality.
